# DDX3 participates in miRNA biogenesis and RNA interference through translational control of PACT and interaction with AGO2

**DOI:** 10.1002/2211-5463.13920

**Published:** 2024-11-14

**Authors:** Ming‐Chih Lai, Yen‐Ling Yu, Chiao‐Nung Chen, Jau‐Song Yu, Hsin‐Yuan Hung, Shih‐Peng Chan

**Affiliations:** ^1^ Department of Biomedical Sciences Chang Gung University Taoyuan Taiwan; ^2^ Graduate Institute of Biomedical Sciences Chang Gung University Taoyuan Taiwan; ^3^ Department of Colorectal Surgery New Taipei Municipal Tucheng Hospital Taiwan; ^4^ Graduate Institute of Microbiology National Taiwan University Taipei Taiwan; ^5^ Molecular Medicine Research Center Chang Gung University Taoyuan Taiwan

**Keywords:** cancer development, DEAD‐box RNA helicase, miRNA biogenesis, RNA interference, translational control

## Abstract

DDX3 is a DEAD‐box RNA helicase that plays multiple roles in RNA metabolism, including translation. We previously reported that DDX3 is required for translation of PACT, a binding partner of Dicer, suggesting a role for DDX3 in microRNA (miRNA) biogenesis and RNA interference (RNAi). Emerging evidence suggests that DDX3 plays a vital role in tumorigenesis and cancer progression, however, its underlying mechanism is still not fully understood. Here, we showed that the control of PACT by DDX3 is conserved in human cells and *Caenorhabditis elegans*. Using a miRNA microarray, we found that DDX3 regulates the expression of a small subset of cancer‐related miRNAs. These oncogenic miRNAs were down‐regulated by knockdown of DDX3 or PACT and up‐regulated by overexpression of DDX3 or PACT in HEK293T cells. Similar results were obtained in human cancer HCT116 and HeLa cells. Dual luciferase reporter assay showed that DDX3 and PACT are required for short hairpin RNA (shRNA)‐induced RNAi. We also performed co‐immunoprecipitation to confirm the interaction between DDX3 and AGO2, a significant component of the RNA‐induced silencing complex, supporting a role for DDX3 in the RNAi pathway. We further examined the effects of DDX3 and PACT on cell proliferation, and stable overexpression of DDX3 in HEK293 cells results in loss of contact inhibition of cell growth. Hence, we propose that DDX3 may participate in cancer development by regulating the RNAi pathway.

## Graphical abstract

DDX3 participates in microRNA (miRNA) biogenesis and RNA interference (RNAi) through translational control of protein activator of the interferon‐induced protein kinase (PACT) and interaction with Argonaute 2 (AGO2). (1) DDX3 regulates the translation of PACT, which is critical for efficient precursor miRNA and short hairpin RNA (shRNA) cleavage. (2) DDX3 associates with AGO2, which is required for RNA‐induced silencing complex (RISC) formation. Therefore, we propose that DDX3 plays a regulatory role in the RNAi pathway.

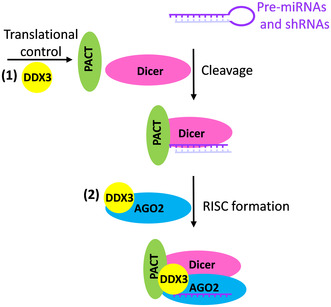

**Abbreviations**
AGO: Argonaute
CRC: colorectal cancer
Fluc: firefly luciferase
GFP: green fluorescent protein
HEK293T: human embryonic kidney 293T
miRNA: microRNA
PACT: protein activator of the interferon‐induced protein kinase
qPCR: quantitative real‐time PCR
RISC: RNA‐induced silencing complex
Rluc: *Renilla* luciferase
RNAi: RNA interference
shRNA: short hairpin RNA
siRNA: small interfering RNA
TRBP: trans‐activation response RNA‐binding protein

Human DDX3 is a member of DEAD‐box RNA helicases that play a crucial role in almost all aspects of RNA metabolism. DDX3 has been implicated in a variety of cellular processes, including transcription [1, 2], RNA transport [3, 4] and translation initiation [5, 6, 7, 8, 9, 10, 11, 12, 13, 14, 15]. We have previously reported that DDX3 is required for translation of mRNAs that contain a long or structured 5′ UTR in cells [6, 7, 12, 13, 14]. Therefore, we propose that DDX3 may facilitate ribosome scanning by resolving mRNA secondary structures during translation initiation. It was recently reported that depletion of DDX3 affects translation of a subset of mRNAs with complex 5′ UTRs [9]. Calviello *et al*. [9] provide evidence that DDX3 binds to helix 16 of the 18S rRNA, and this site is adjacent to the mRNA entry channel of the small ribosomal subunit. These findings provide a mechanistic explanation for the role of DDX3 in translation initiation.

DDX3 is involved in a variety of biological functions, including cell cycle [7, 16], innate immunity [12, 13, 17, 18, 19] and cancer development [20, 21]. The role of DDX3 in cancer development has attracted more attention in recent years. However, it is a complicated and controversial issue. DDX3 may act as either an oncogene or a tumor suppressor gene in different types of cancer [22]. Most clinical studies suggest that DDX3 plays an oncogenic role in many cancers, including breast cancer [23, 24], lung cancer [25], colorectal cancer [21, 26, 27] and Ewing sarcoma [28]. We previously found that DDX3 regulates the cell cycle through translational control of cyclin E1 in HeLa cells [7], supporting an oncogenic role for DDX3 in cancer development. RK‐33 is a small‐molecule and selective DDX3 inhibitor [25]. It has been reported that treatment with RK‐33 causes cell cycle arrest at the G1 phase in different cancer cells. Therefore, DDX3 has been considered as a potential therapeutic target in cancer treatment [25, 26, 29].

There is some evidence that DDX3 functions in the RNA interference (RNAi) pathway [30, 31, 32, 33, 34, 35]. The RNAi pathway is an evolutionarily conserved gene‐silencing process triggered by two types of small RNA molecules: microRNA (miRNA) and small interfering RNA (siRNA). miRNAs are small non‐coding RNA molecules that regulate gene expression, particularly during embryonic development. In the nucleus, the primary miRNAs are transcribed by RNA polymerase II or III and cleaved by the RNase III enzyme Drosha to produce ~70‐nt hairpin precursor miRNAs (pre‐miRNAs) [36]. The precursor miRNAs are exported to the cytoplasm and cleaved by the RNase III enzyme Dicer to generate mature miRNAs. Mature miRNAs are assembled into the RNA‐induced silencing complex (RISC), which mediates the silencing of complementary target mRNAs [36]. The Argonaute (AGO) protein family is the catalytic core of the RISC [37]. miRNAs interact with AGO proteins and guide them to silence target mRNAs through translational repression or mRNA degradation [38, 39]. DDX3 has been identified as a component of the primary miRNA processing complex [35], implying a role for DDX3 in miRNA biogenesis. A genome‐wide screen of the components required for RNAi in *Caenorhabditis elegans* identified Y38A10A.6, a homolog of DDX3, as an indispensable gene [40]. In *Drosophila*, DDX3 homolog Belle has been validated as a bona fide component of the RNAi pathway [32]. It has been reported that Belle/DDX3 and the RNAi pathway play a similar role in regulating chromosome segregation in *Drosophila* and human cells [33]. Notably, the interaction between Belle/DDX3 and AGO proteins was revealed by co‐immunoprecipitation experiments in *Drosophila* S2 cells [32, 33]. DDX3 has been identified as an essential factor in the mammalian RNAi pathway using a short hairpin RNA (shRNA)‐expression library [31]. DDX3 was shown to co‐localize with AGO2 in the cytoplasm of HeLa S3 cells [31]. These findings suggest that DDX3 may play a vital role in the RNAi pathway.

We previously reported that DDX3 is required for translation of the protein activator of the interferon‐induced protein kinase (PACT) in human cells [13]. PACT is a double‐stranded RNA‐binding protein that facilitates the recognition of viral RNAs by retinoic acid‐inducible gene‐I‐like receptors [13] and double‐stranded RNA‐activated protein kinase [41] during antiviral immune responses. PACT and trans‐activation response RNA‐binding protein (TRBP) also bind to Dicer and facilitate substrate cleavage specificity during miRNA biogenesis [42]. Depletion of PACT affects the accumulation of mature miRNA *in vivo* and moderately reduces the efficiency of siRNA‐induced RNAi [43]. We therefore propose that DDX3 may function in the RNAi pathway through translational control of PACT and interaction with AGO2. The control of RNAi may contribute to the oncogenic or tumor‐suppressive functions of DDX3 in cancer development.

## Materials and methods

All materials were purchased from Sigma‐Aldrich (St Louis, MO, USA) unless otherwise specified.

### Cell culture and transfection

Human embryonic kidney 293T (HEK293T), HCT116 and HeLa cell lines were purchased from the American Type Culture Collection (ATCC Manassas, VA, USA) and cultured in high‐glucose Dulbecco's modified Eagle's medium supplemented with 10% fetal bovine serum, 100 U·mL^−1^ penicillin, 100 μg·mL^−1^ streptomycin and 2 mm l‐glutamine at 37 °C in 5% CO_2_ incubator. Transfection was performed using the Lipofectamine® 2000 (Thermo Fisher Scientific, Waltham, MA, USA) in accordance with the manufacturer's instructions.

### Lentivirus‐mediated RNAi knockdown

The National RNAi Core Facility (Academia Sinica, Taiwan) provided all of the plasmids required to produce lentivirus. The pLKO.1 vectors expressing shRNAs were: shDDX3‐1 (TRCN0000000002), shDDX3‐2 (TRCN0000000004), shFluc (TRCN0000231693), shPACT‐1 (TRCN0000196873) and shPACT‐2 (TRCN0000052647). A packaging vector (pCMVΔR8.91), an envelope vector (pMD.G) and a pLKO.1‐based shRNA vector were co‐transfected into HEK293T cells for lentiviral production. To knockdown endogenous DDX3 or PACT, cells were transduced with shRNA‐expressing lentivirus at a multiplicity of infection of five particles per cell in Dulbecco's modified Eagle's medium containing 8 μg·mL^−1^ polybrene at 37 °C and 5% CO_2_. After 24 h, 2 μg·mL^−1^ puromycin was added to the medium for selecting infected cells, except HEK293T. Cells were harvested 3 days after transduction for analysis.

### RK‐33 treatment

RK‐33 (S8246‐2mg) was purchased from Selleckchem (Houston, TX, USA). A stock solution of 10 mm in 100% dimethyl sulfoxide was prepared. For inhibition of the helicase activity of DDX3, HEK293T cells were treated with 2 μm RK‐33 for 24 h.

### Western blot analysis

Proteins were transferred onto a poly(vinylidene difluoride) transfer membrane (Millipore, Billerica, MA, USA). Protein blots were blocked by 3% skim milk in TBST (100 mm Tris‐HCl, pH 7.6, 150 mm NaCl and 0.1% Tween 20) at room temperature for 1 h. The primary antibodies used included affinity‐purified rabbit anti‐DDX3 (0.1 μg·mL^−1^), rabbit anti‐PACT (dilution 1 : 1000, #13490; Cell Signaling Technology, Danvers, MA, USA), rabbit anti‐TRBP (0.5 μg·mL^−1^, ab42018; Abcam, Cambridge, UK), mouse anti‐α‐tubulin (0.2 μg·mL^−1^, sc‐32293; Santa Cruz Biotechnology, Santa Cruz, CA, USA), rabbit anti‐VBH‐1 antibody (dilution 1 : 1000, custom antibody; GenScript, Piscataway, NJ, USA), rabbit anti‐ green fluorescent protein (GFP) (0.2 μg·mL^−1^, ab290; Abcam), mouse anti‐β‐actin (0.2 μg·mL^−1^, sc‐47778; Santa Cruz Biotechnology), mouse anti‐AGO2 antibody (dilution 1 : 1000, MA523515; Invitrogen, Waltham, MA, USA), rabbit anti‐Dicer antibody (dilution 1 : 1000, GTX130536; GeneTex, Irvine, CA, USA) and rabbit anti‐FLAG antibody (dilution 1 : 1000, #14793; Cell Signaling Technology). Protein blots were incubated with primary antibodies in a blocking buffer at room temperature for 2 h, followed by horseradish peroxidase (HRP)‐conjugated secondary antibodies at room temperature for 2 h. Signals were detected using Immobilon Western Chemiluminescent HRP Substrate (Millipore) and images from autoradiograms were captured with the ChemiDoc™ Touch Imaging System (Bio‐Rad, Hercules, CA, USA).

### Plasmid construction

The pcDNA3.1 plasmids expressing FLAG‐tagged DDX3 (FLAG‐DDX3) and PACT (PACT‐FLAG) have been described previously [2, 13]. The plasmids expressing GFP (pEGFP‐C1) and GFP‐DDX3 have also been described previously [6].

### Generation of stable cell lines

To establish stable HEK293 cell lines that overexpress DDX3, HEK293 cells were transfected with the pcDNA3.1 plasmid expressing FLAG‐DDX3 and selected with G418 (350 μg·mL^−1^) for 2–3 weeks. G418‐resistant colonies were picked and seeded into 24‐well plates containing complete medium supplemented with G418 for 2–3 weeks. The surviving colonies were screened for stable overexpression of FLAG‐DDX3 protein by western blot analysis.

### Gene knockdown in *C. elegans*

Two days before the experiments, freshly grown HT115 bacteria containing the empty vector L4440 or RNAi plasmids were seeded on RNAi nematode growth media plates supplemented with 100 μg·mL^−1^ ampicillin and 1 mm isopropyl thio‐β‐d‐galactoside. Primer sets for RNAi clones are listed in Table 1. *C. elegans* were synchronized by standard bleaching procedure, and the yielding eggs were left to hatch in M9 buffer at 20 °C. After 18 h of hatching, ~500 synchronized L1 worms were pipetted onto plates containing RNAi bacteria and transferred to 20 °C. After 48 h, worms were harvested for subsequent assays.

**Table 1:** List of primers used for RNAi clones in *C. elegans.*

	Primer name	Primer sequence (5′‐ to 3′)
*vbh‐1*	Forward primer	TCGTTGGCTCCTCTCTTTGT
	Reverse primer	CCGAGAGTAATCAATGGGGA
*rde‐12*	Forward primer	CTTTGACCTTCCTGACGGAG
	Reverse primer	ACGGGGATACGGTGGAATA
*rde‐4*	Forward primer	CACTGTTTAGCCTCTTCCGC
	Reverse primer	CTGTCGAACTTCCTGAAGGC

### RNA isolation

Total RNA from cells was extracted using TRIzol Reagent (Thermo Fisher Scientific) in accordance with the manufacturer's instructions. Genomic DNA was removed using the TURBO DNA‐free Kit (Thermo Fisher Scientific). RNA concentration was determined using NanoDrop 2000 spectrophotometer (Thermo Fisher Scientific).

### miRNA quantitative real‐time PCR (qRT‐PCR)

Total RNA extracted from cells was treated with the TURBO DNA‐free kit (Thermo Fisher Scientific) and then reverse‐transcribed into cDNA using M‐MLV Reverse Transcriptase (Thermo Fisher Scientific) and miRNA‐specific RT primers. According to the supplier's recommendations, the resulting cDNA was subjected to qRT‐PCR analysis using StepOnePlus Real‐Time PCR System (Thermo Fisher Scientific). The expression level of miRNAs was detected using Fast SYBR Green Master Mix (Thermo Fisher Scientific) and miRNA‐specific PCR primers (Table 2). Quantitative analysis measured cycle threshold (*C*_t_) values during the exponential amplification phase. Relative quantitation values were calculated using the $2^{-\Delta \Delta C_{t}}$ method. miRNA expression was normalized to U6 snRNA levels.

**Table 2:** List of miRNA primers used for quantitative PCR.

miRNA	Primer name	Primer sequence (5′‐ to 3′)
miR‐18a	RT‐primer	CTCAACTGGTGTCGTGGAGTCGGCAATTCAGTTGAGCTATCTGC
	Forward primer	CGGCGGTAAGGTGCATCTAGTG
miR‐20b	RT‐primer	CTCAACTGGTGTCGTGGAGTCGGCAATTCAGTTGAGCTACCTGC
	Forward primer	CGGCGGCAAAGTGCTCATAGTG
miR‐93	RT‐primer	CTCAACTGGTGTCGTGGAGTCGGCAATTCAGTTGAGCTACCTGC
	Forward primer	CGGCGGCAAAGTGCTGTTCGTG
miR‐221	RT‐primer	CTCAACTGGTGTCGTGGAGTCGGCAATTCAGTTGAGGAAACCCA
	Forward primer	CGGCGGAGCTACATTGTCTGCT
miR‐222	RT‐primer	CTCAACTGGTGTCGTGGAGTCGGCAATTCAGTTGAGACCCAGTA
	Forward primer	CGGCGGAGCTACATCTGGCTAC
miR‐320a	RT‐primer	CTCAACTGGTGTCGTGGAGTCGGCAATTCAGTTGAGTCGCCCTC
	Forward primer	CGGCGGAAAAGCTGGGTTGAGA
miR‐619	RT‐primer	CTCAACTGGTGTCGTGGAGTCGGCAATTCAGTTGAGGGCTCATG
	Forward primer	CGGCGGGCTGGGATTACAGGCA
miR‐4448	RT‐primer	CTCAACTGGTGTCGTGGAGTCGGCAATTCAGTTGAGTACCCCTA
	Forward primer	CGGCGGGGCTCCTTGGTCTAGG
miRNA	Reverse primer	CTGGTGTCGTGGAGTCGGCAATTC
U6	Forward primer	CTCGCTTCGGCAGCACA
	Reverse primer	AACGCTTCACGAATTTGCGT

### Dual luciferase reporter assay

Cells (4 × 10^4^ cells per well/24‐well plate) were transduced with the empty lentiviral vector pLKO.1 or the pLKO.1 vector expressing shDDX3 or shPACT, respectively. At 2 days after transduction, cells were co‐transfected with the firefly luciferase (Fluc) reporter (pFL‐SV40), the pRL‐SV40 vector and the pLKO.1 vector expressing shFluc. At 24 h after transfection, cells were lysed in 1× Passive Lysis Buffer (Promega, Madison, WI, USA). The activities of Fluc and *Renilla* luciferase (Rluc) were measured using a Dual‐Luciferase Reporter Assay System (Promega) and a GloMax 20/20 Luminometer (Promega).

### Immunoprecipitation

GFP‐DDX3 was expressed in HEK293T cells for 2 days. HEK293T cells (1 × 10^7^) were lysed in 500 μL of RIPA buffer (25 mm Tris‐HCl, pH 7.5, 150 mm NaCl) containing 1% NP‐40 and 1× protease inhibitor cocktail (Thermo Fisher Scientific). To immunoprecipitate GFP‐DDX3, 2 μL of anti‐GFP antibody (ab290; Abcam) were coupled to 10 μL of nProtein A Sepharose 4 Fast Flow (GE Healthcare, Chicago, IL, USA) beads in RIPA buffer (25 mm Tris‐HCl, pH 7.5, 150 mm NaCl) containing 0.1% NP‐40. Cell lysates (1 mg) were incubated with the antibody beads at 4 °C with gentle agitation for 2 h. The beads were washed four times with 1 mL of cold RIPA buffer containing 0.1% NP‐40 to remove unbound proteins. Immunoprecipitates were treated with 1 mg·mL^−1^ RNase A at 37 °C for 30 min. Bound proteins were extracted with 1× SDS sample buffer and resolved on SDS/PAGE, followed by western blot analysis. To immunoprecipitate endogenous AGO2, 4 μL of anti‐AGO2 antibody (MA523515; Invitrogen) were coupled to 10 μL of nProtein A Sepharose 4 Fast Flow (GE Healthcare) beads. To immunoprecipitate FLAG‐tagged DDX3 and PACT, FLAG‐tagged proteins were transiently expressed in HEK293T cells for 2 days. Cell lysates were incubated with 10 μL of ANTI‐FLAG M2 Affinity Gel (A2220; Millipore) at 4 °C with gentle agitation for 2 h.

### Cell proliferation/viability assay

Cell viability was determined using the CellTiter 96 AQueous One Solution Cell Proliferation Assay (Promega) in accordance with the manufacturer's instructions. Briefly, 3 × 10^3^ cells were allowed to attach overnight in a 96‐well plate. Viable cells per well were estimated every 24 h after 1, 2 and 3 days of growth. Cells were incubated with MTS reagent for 1 h, after which absorbance was measured at 490 nm with the SpectraMax M2 Multimode Plate Reader (Molecular Devices, San Jose, CA, USA).

### Statistical analysis

All results are expressed as the mean ± SD from at least three independent experiments. Statistical analysis was performed using an unpaired Student's *t*‐test. *P* < 0.05 was considered statistically significant.

## Results

### DDX3‐mediated translational control of PACT in human cells and *C. elegans*

**Fig. 1:**
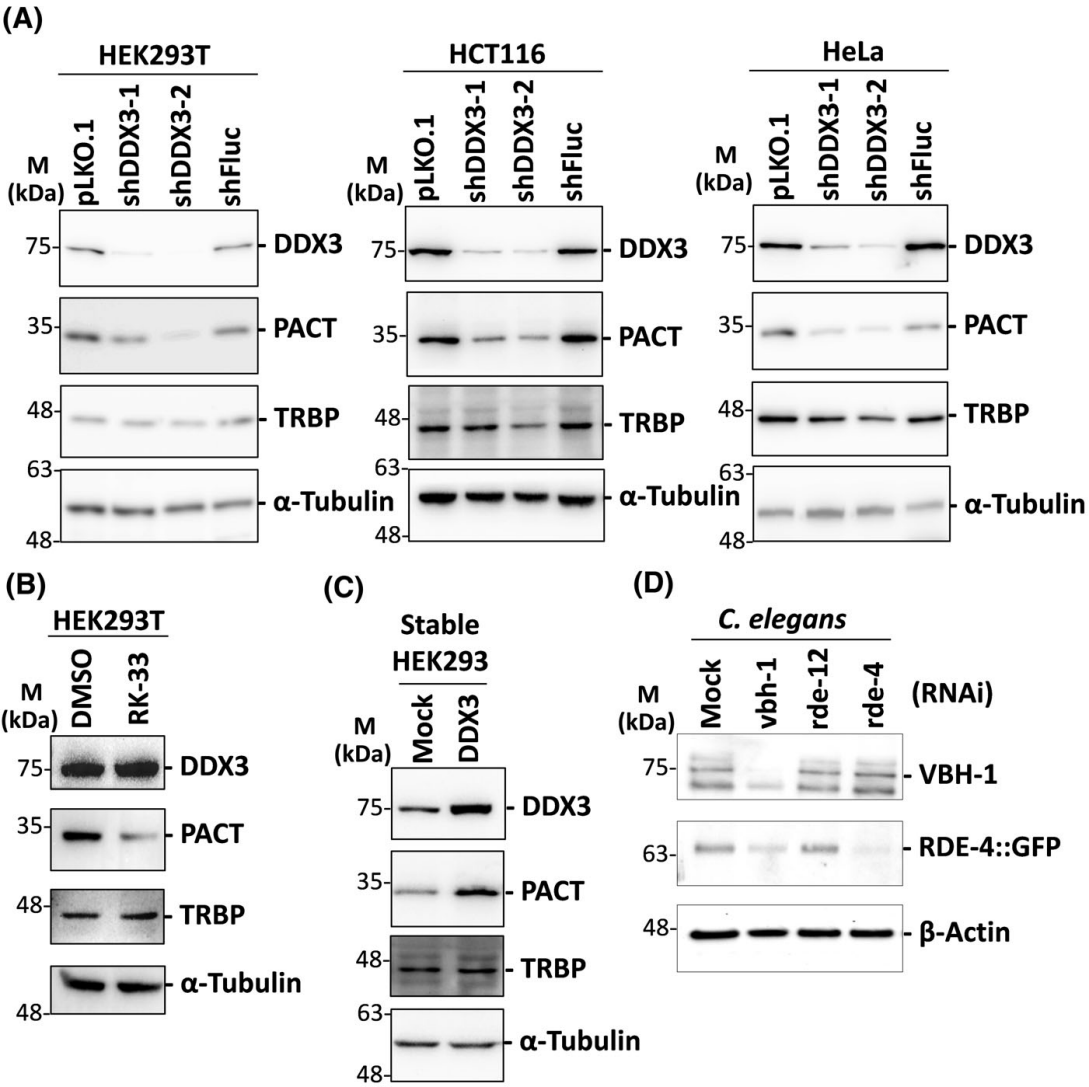
DDX3 specifically regulates the expression of PACT in human cells and *C. elegans*. (A) HEK293T, HeLa and HCT116 cells were transduced with the empty lentiviral vector (pLKO.1) or the pLKO.1 vector expressing the indicated shRNAs. Cells were harvested for analysis 3 days after transduction. Western blot analysis was performed with antibodies against DDX3, PACT, TRBP and α‐tubulin (*n* = 3). The detection of α‐tubulin was used as a loading control. (B) HEK293T cells were treated with dimethyl sulfoxide or RK‐33 for 24 h. Cells were harvested for western blot analysis with antibodies against DDX3, PACT, TRBP and α‐tubulin (*n* = 2). The detection of α‐tubulin was used as a loading control. (C) A stable HEK293 cell line that overexpresses DDX3 was established. Total cell extracts prepared from DDX3‐overexpressing HEK293 cells were subjected to western blot analysis with antibodies against DDX3, PACT, TRBP and α‐tubulin (*n* = 2). The detection of α‐tubulin was used as a loading control. (D) Transgenic *C. elegans* C860 were fed with the various RNAi bacteria for gene silencing. Total worm extracts were prepared from RDE‐4‐GFP‐expressing *C. elegans* and subjected to western blot analysis with antibodies against VBH‐1, GFP and β‐actin (*n* = 2). The detection of β‐actin was used as a loading control.

We previously reported that DDX3 is required for translation of PACT mRNA in human cells [13]. To confirm the results, we performed lentivirus‐mediated RNAi knockdown of DDX3 in human embryonic kidney 293T (HEK293T) cells, colorectal carcinoma HCT116 cells and cervical adenocarcinoma HeLa cells. Western blot analysis showed that the expression of DDX3 protein was down‐regulated by two DDX3‐targeting shRNAs (shDDX3‐1 and shDDX3‐2), and shDDX3‐2 was more effective than shDDX3‐1 in inhibiting the expression of DDX3 (Fig. 1A). An shRNA against firefly luciferase (shFluc) was used as a negative control. After normalization to α‐tubulin, the level of PACT protein had declined considerably in DDX3 knockdown cells compared to control cells, suggesting that DDX3 controls the expression of PACT (Fig. 1A). Compared to PACT, the expression of another Dicer partner TRBP was only slightly down‐regulated by DDX3 knockdown (Fig. 1A). This indicates that DDX3‐mediated translational control of PACT is specific. RK‐33 is a small molecule inhibitor that specifically inhibits the helicase activity of DDX3 [25]. RK‐33 treatment did not affect the expression of DDX3 but decreased the protein level of PACT (Fig. 1B). Therefore, the helicase activity of DDX3 is required for the expression of PACT. We also detected the expression of PACT and TRBP proteins in HEK293 cells that stably overexpress DDX3. By contrast, the protein level of PACT rather than TRBP was up‐regulated by DDX3 overexpression (Fig. 1C). We therefore conclude that DDX3 highly regulates PACT in human cells. To examine whether the control of PACT by DDX3 is evolutionarily conserved, we used double‐stranded RNA‐induced RNAi to knock down two homologs of human DDX3 in *C. elegans*, VBH‐1 and RDE‐12 [30, 32]. The results showed that the expression of RDE‐4, the homolog of human PACT in *C. elegans*, is also down‐regulated by VBH‐1 knockdown (Fig. 1D). By contrast, the expression of RDE‐4 protein was not affected by depletion of another DDX3 homolog, RDE‐12, in *C. elegans*. This result suggests that RDE‐12 is not functionally the same as human DDX3. RDE‐12 strongly resembles Vasa/DDX4, whereas VBH‐1 is closely related to Belle/DDX3 [30]. Altogether, DDX3‐mediated translational control of PACT may be evolutionarily conserved from *C. elegans* to humans.

### Depletion of DDX3 affects the expression of a small subset of cancer‐related miRNAs in HEK293T cells

To identify miRNAs regulated by DDX3, we used OneArray miRNA Microarrays (Phalanx Biotech Group, Hsinchu, Taiwan) to screen differentially expressed miRNAs in DDX3 knockdown HEK293T cells compared to control cells. A volcano plot shows the relationship between fold change and statistical significance (Fig. S1). The results from two independent microarray experiments identified 12 miRNAs expressed at significantly different levels (*P* < 0.05). Among these candidate miRNAs, 10 were down‐regulated (Table 3) and two were up‐regulated (Table 4) in DDX3 knockdown HEK293T cells compared to mock‐treated cells. The two up‐regulated miRNAs were miR‐6890 and miR‐1246 (Table 4). The function of miR‐6890 still needs to be determined. However, the role of miR‐1246 in the carcinogenesis of different cancers is obscure [44]. We therefore focused on down‐regulated candidate miRNAs in DDX3 knockdown HEK293T cells compared to control cells. These 10 down‐regulated candidate miRNAs, including miR‐20b, miR‐18a, miR‐18b, miR‐222, miR‐221, miR‐93, miR‐320a, miR‐320b, miR‐320d and miR‐320e (Table 3), can be divided into three miRNA gene families (miR‐17, miR‐221 and miR‐320). miRNAs may function as an oncogene or a tumor suppressor gene in cancer development, depending on its target mRNAs. Most of the miR‐17 family miRNAs (e.g. miR‐18a/b, miR‐20b and miR‐93) [45, 46, 47, 48, 49, 50, 51, 52, 53, 54] and the miR‐221 family miRNAs (e.g. miR‐221 and miR‐222) [55, 56, 57] have been implicated as oncogenic miRNAs that are associated with carcinogenesis, malignant transformation and metastasis. By contrast, the miR‐320 family miRNAs (e.g. miR‐320a and miR‐320b) are considered to be tumor suppressor genes, which serve as a repressor of tumor proliferation, metastasis and epithelial–mesenchymal transition [58, 59, 60]. The results of the miRNA microarray analysis suggest that DDX3 may function as an oncogene or a tumor suppressor gene by regulating the expression of a small subset of miRNAs.

**Table 3:** List of 10 candidate miRNAs for which expression is down‐regulated in DDX3 knockdown HEK293T cells compared to mock‐treated cells.

miRNA	*P*‐value	Mock control	DDX3 knockdown	Fold change
has‐miR‐20b‐5p	0.0000976	359	229	0.64
has‐miR‐18a‐5p	0.000236353	435	250	0.57
has‐miR‐18b‐5p	0.001307906	287	189	0.66
has‐miR‐222‐3p	0.001516814	449	220	0.49
has‐miR‐221‐3p	0.001698622	896	447	0.49
has‐miR‐320a	0.005139361	551	337	0.61
has‐miR‐93‐5p	0.005614619	537	338	0.63
has‐miR‐320b	0.010523447	500	314	0.63
has‐miR‐320d	0.013752286	343	196	0.57
has‐miR‐320e	0.044844237	270	178	0.66

**Table 4:** List of two candidate miRNAs for which expression is up‐regulated in DDX3 knockdown HEK293T cells compared to mock‐treated cells.

miRNA	*P*‐value	Mock control	DDX3 knockdown	Fold change
has‐miR‐6890‐5p	0.03477611	437	816	1.87
has‐miR‐1246	0.047224725	34 843	52 719	1.51

### Validation of candidate miRNAs regulated by DDX3 in HEK293T cells

**Fig. 2:**
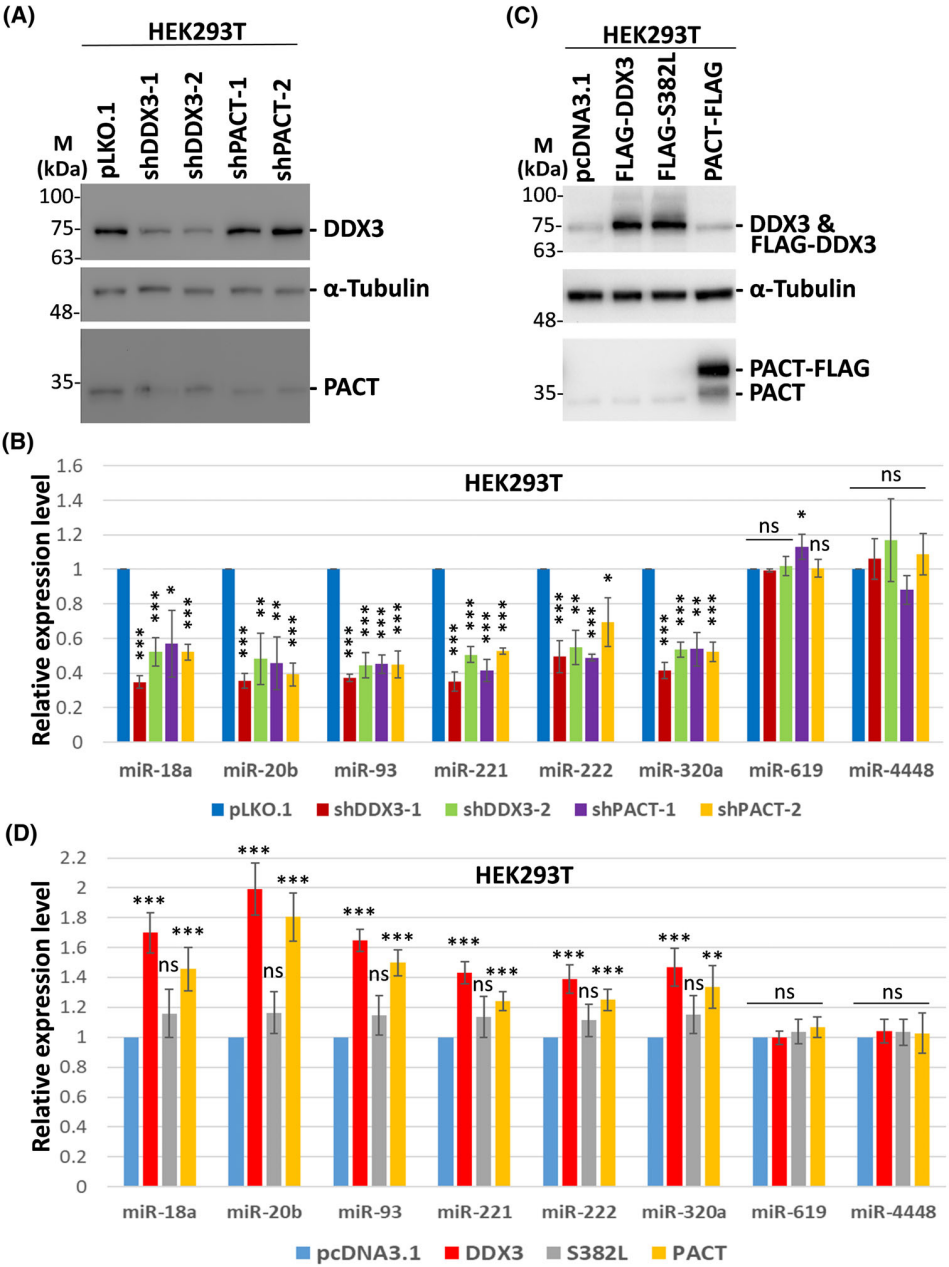
Selective miRNAs are regulated by DDX3 and PACT in HEK293T cells. (A) HEK293T cells were transduced with the empty lentiviral vector (pLKO.1) or the pLKO.1 vector expressing the indicated shRNAs. Cells were harvested for analysis 3 days after transduction. Western blot analysis was performed with antibodies against DDX3, α‐tubulin and PACT (*n* = 3). The detection of α‐tubulin was used as a loading control. (B) HEK293T cells were transduced with the empty lentiviral vector (pLKO.1) or the pLKO.1 vector expressing the indicated shRNAs. Total RNA was extracted using the TRIzol reagent 3 days after transduction. miRNAs were analyzed by qRT‐PCR. Bar graphs show the relative expression levels of miRNAs normalized to U6 snRNA. Data are shown as the mean ± SD from three independent experiments. (C) HEK293T cells were transiently transfected with the pcDNA3.1 vector or the pcDNA3.1 vector expressing FLAG‐tagged DDX3, DDX3 mutant (S382L) or PACT. Cells were harvested for analysis 2 days after transfection. Western blot analysis was performed with antibodies against DDX3, α‐tubulin and PACT (*n* = 3). The detection of α‐tubulin was used as a loading control. (D) HEK293T cells were transiently transfected with the pcDNA3.1 vector or the pcDNA3.1 vector expressing FLAG‐tagged DDX3, DDX3 mutant (S382L) or PACT. Total RNA was extracted using the TRIzol reagent 2 days after transfection. miRNAs were analyzed by qRT‐PCR. Bar graphs show the relative expression levels of miRNAs normalized to U6 snRNA. Data are shown as the mean ± SD from three independent experiments. Statistical analyses were carried out using Student's *t* test (ns, not significant; *P < 0.05; **P < 0.01; ***P < 0.001).

To validate these down‐regulated candidate miRNAs (Table 3), we performed lentivirus‐mediated RNAi knockdown of DDX3 or PACT in HEK293T cells and then used qRT‐PCR analysis to detect the expression of specific miRNAs. Western blot analysis showed that the protein level of DDX3 or PACT was obviously reduced in HEK293T cells transduced with DDX3‐targeting shRNAs (shDDX3‐1 and shDDX3‐2) or PACT‐targeting shRNAs (shPACT‐1 and shPACT‐2), respectively (Fig. 2A). The expression levels of miR‐18a, miR‐20b, miR‐93, miR‐221, miR‐222 and miR‐320a were clearly down‐regulated by DDX3 or PACT knockdown in HEK293T cells (Fig. 2B), suggesting that DDX3 and PACT synchronously regulate these candidate miRNAs. By contrast, miR‐619 and miR‐4448, which were not affected by DDX3 knockdown in the results of miRNA microarray analysis, served as negative controls. To further confirm these observations, HEK293T cells were transiently transfected with a plasmid that overexpresses DDX3 or PACT protein (Fig. 2C). The expression levels of miR‐18a, miR‐20b, miR‐93, miR‐221, miR‐222 and miR‐320a were up‐regulated by DDX3 or PACT overexpression in HEK293T cells (Fig. 2D). This result reinforced the role of DDX3 and PACT in regulating the expression of specific miRNAs. We also evaluated whether the RNA helicase activity of DDX3 plays a role in miRNA induction. A helicase‐defective mutant (S382L) of DDX3 was transiently expressed in HEK293T cells (Fig. 2C). The results showed that overexpression of wild‐type DDX3, but not the S382L mutant, induced these candidate miRNAs (Fig. 2D). Therefore, the RNA helicase activity of DDX3 is critical for its function in regulating miRNAs.

### DDX3 and PACT regulate candidate miRNAs in human cancer cells

**Fig. 3:**
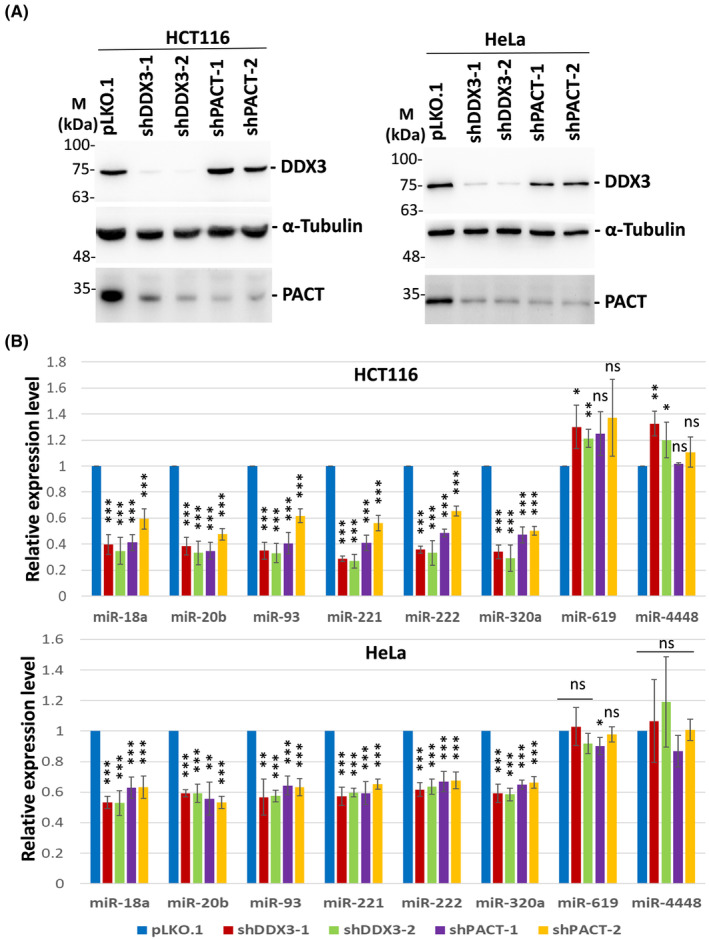
Selective miRNAs are regulated by DDX3 and PACT in HCT116 and HeLa cells. (A) HCT116 and HeLa cells were transduced with the empty lentiviral vector (pLKO.1) or the pLKO.1 vector expressing the indicated shRNAs. Cells were harvested for analysis 3 days after transduction. Western blot analysis was performed with antibodies against DDX3, α‐tubulin and PACT (*n* = 3). The detection of α‐tubulin was used as a loading control. (B) HCT116 and HeLa cells were transduced with the empty lentiviral vector (pLKO.1) or the pLKO.1 vector expressing the indicated shRNAs. After 24 h, 2 μg·mL^−1^ puromycin was added to the medium for selecting infected cells. Total RNA was extracted using the TRIzol reagent 3 days after transduction. miRNAs were analyzed by qRT‐PCR. Bar graphs show the relative expression levels of miRNAs normalized to U6 snRNA. Data are shown as the mean ± SD from three independent experiments. Statistical analyses were carried out using Student's *t* test (ns, not significant; *P < 0.05; **P < 0.01; ***P < 0.001).

To determine whether DDX3 and PACT regulate candidate miRNAs in human cancer cells, we performed lentivirus‐mediated RNAi knockdown of DDX3 or PACT in HCT116 and HeLa cells. After lentiviral transduction and puromycin selection, DDX3 and PACT proteins were dramatically reduced by shDDX3 and shPACT, respectively (Fig. 3A). The decrease in the protein level of PACT caused by DDX3 knockdown is almost equivalent to that of PACT knockdown in HCT116 and HeLa cells, suggesting that DDX3 highly regulates PACT in human cancer cells. We also used qRT‐PCR analysis to detect the expression of candidate miRNAs in HCT116 and HeLa cells. The pattern of down‐regulated miRNAs was consistent with that observed in DDX3 knockdown HEK293T cells compared to mock‐treated cells (Fig. 3B). Therefore, DDX3 may function as an oncogene or a tumor suppressor gene by regulating the expression of cancer‐related miRNAs in human cells.

### DDX3 and PACT are required for shRNA‐induced gene silencing

**Fig. 4:**
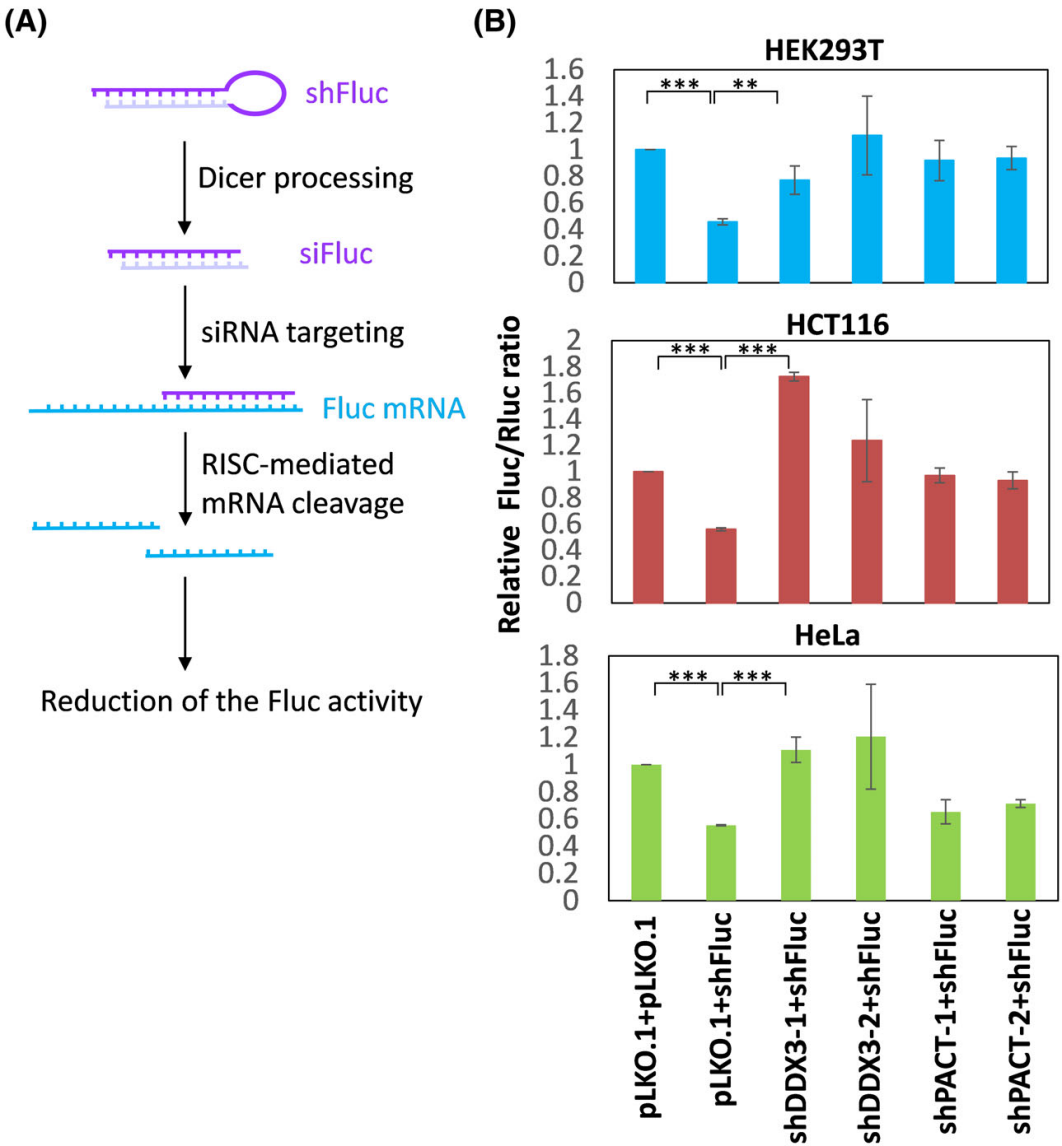
DDX3 and PACT are required for shRNA‐induced RNAi. (A) Schematic representation of the strategy for measuring the gene‐silencing activity of shFluc. (B) HEK293T, HCT116 and HeLa cells were transduced with the empty lentiviral vector (pLKO.1) or the pLKO.1 vector expressing shDDX3 or shPACT. After 24 h, cells were transiently transfected with the pLKO.1 vector or the pLKO.1 vector expressing shFluc together with the firefly luciferase (Fluc) and *Renilla* luciferase (Rluc) reporter constructs. Cells were lysed for dual‐luciferase reporter assay 3 days after transduction. For each transfectant, the Fluc activity was normalized to that of the Rluc control. Bar graphs show the relative Fluc/Rluc ratio in DDX3‐ or PACT‐depleted cells compared to mock‐treated (pLKO.1) cells. Data are shown as the mean ± SD from three independent experiments. Statistical analyses were carried out using Student's *t* test (**P < 0.01; ***P < 0.001).

We next examined whether DDX3 and PACT are involved in the RNAi response induced by shRNAs. To measure the gene‐silencing activities of shRNAs, we employed a firefly luciferase (Fluc) mRNA‐targeting shRNA (shFluc), which is processed into a Fluc‐specific siRNA (siFluc) and then triggers cleavage and degradation of Fluc mRNA (Fig. 4A). If DDX3 or PACT is critical for RNAi, shFluc‐induced gene silencing would be less effective in DDX3 or PACT knockdown cells. The pLKO.1 plasmid expressing shFluc was co‐transfected with the Fluc and Rluc reporter constructs into DDX3 or PACT knockdown cells. The empty shRNA expression plasmid pLKO.1 was used as a negative control. The gene‐silencing activity of shFluc was determined by calculating the ratio of Fluc activity normalized to Rluc (Fluc/Rluc). The results of the dual luciferase reporter assay showed that knockdown of DDX3 or PACT significantly decreases shFluc‐induced gene silencing in HEK293T, HCT116 and HeLa cells (Fig. 4B), indicating that DDX3 and PACT are required for shRNA‐induced RNAi. Compared to HEK293T and HCT116 cells, RNAi was only slightly affected by PACT knockdown in HeLa cells (Fig. 4B). This result is consistent with a moderate decrease of miRNA expression in PACT knockdown HeLa cells (Fig. 3B). However, the regulation and function of RNAi may vary in different cell types.

### DDX3 interacts with AGO2 in HEK293T cells

**Fig. 5:**
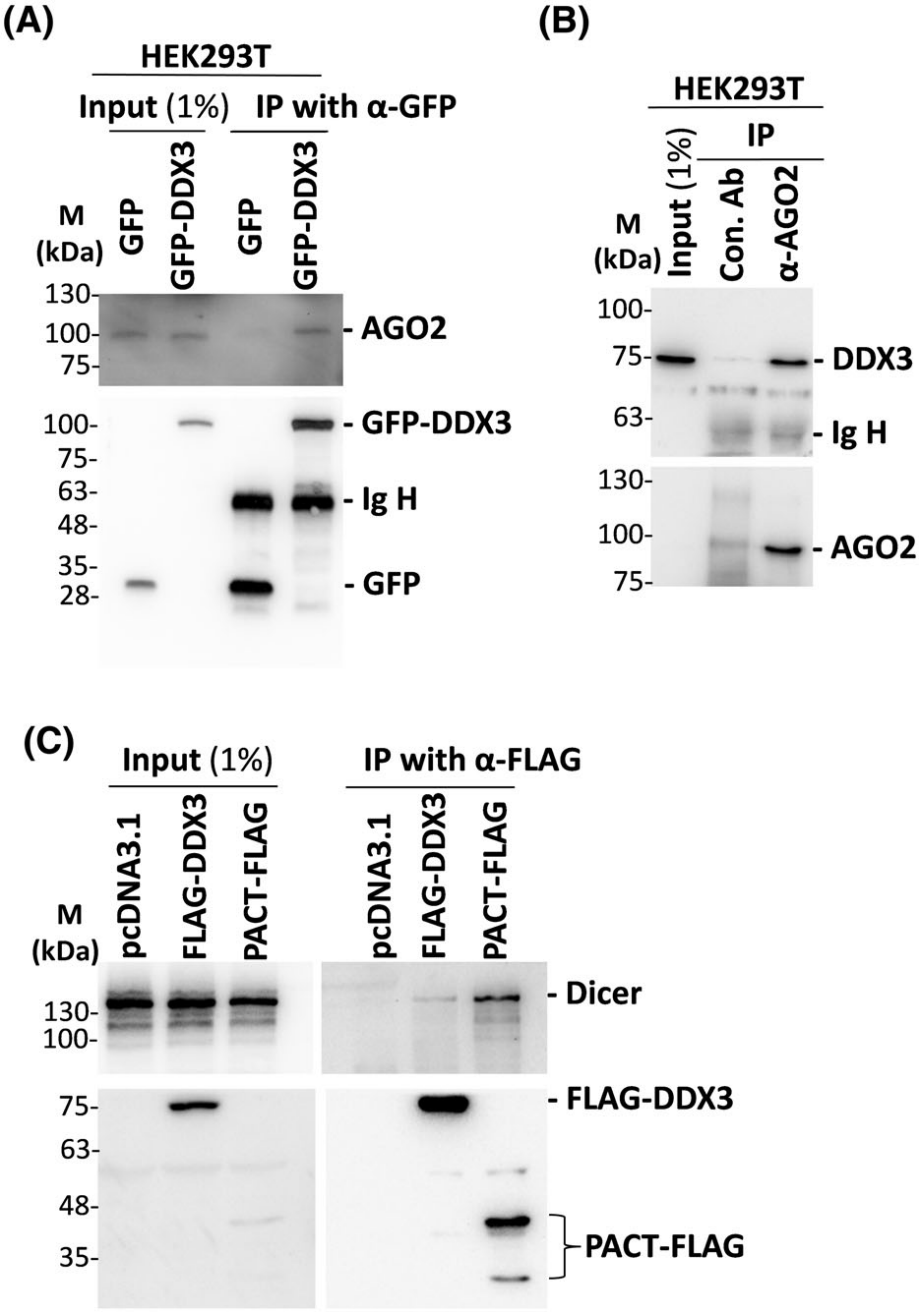
DDX3 is associated with AGO 2 and Dicer in HEK293T cells. (A) HEK293T cells (5 × 10^6^) were transiently transfected with the pEGFP‐C1 vector (GFP) or the pEGFP‐C1 vector expressing GFP‐DDX3. After 24 h, cells were harvested for immunoprecipitation with an anti‐GFP antibody. Co‐precipitated proteins were resolved on 10% SDS/PAGE, followed by western blot analysis with anti‐AGO2 and anti‐GFP antibodies (*n* = 3). Ig H represents the immunoglobulin heavy chain. (B) HEK293T cells (1 × 10^7^) were harvested for immunoprecipitation with anti‐AGO2 or mouse IgG control antibody. Co‐precipitated proteins were resolved on 8% SDS/PAGE, followed by western blot analysis with anti‐DDX3 and anti‐AGO2 antibodies (*n* = 2). Ig H represents the immunoglobulin heavy chain. (C) HEK293T cells (5 × 10^6^) were transiently transfected with the pcDNA3.1 vector or the pcDNA3.1 vector expressing FLAG‐tagged DDX3 or PACT. After 24 h, cells were harvested for immunoprecipitation with an anti‐FLAG antibody. Co‐precipitated proteins were resolved on 8% SDS/PAGE, followed by western blot analysis with anti‐Dicer and anti‐FLAG antibodies (*n* = 2).

Co‐immunoprecipitation experiments indicated that Belle/DDX3 interacts with AGO2 protein in *Drosophila* S2 cells [33]. Therefore, we also examined whether DDX3 interacts with AGO2 in human cells. GFP‐DDX3 was transiently expressed in HEK293T cells, and cell lysates were prepared and subjected to immunoprecipitation with an anti‐GFP antibody. The results of western blot analysis showed that approximately 1.2% of endogenous AGO2 protein was co‐precipitated with GFP‐DDX3 protein (Fig. 5A). AGO2 protein was co‐precipitated with GFP‐DDX3 after the treatment with RNase A, suggesting a direct interaction between DDX3 and AGO2. Conversely, immunoprecipitation experiments were performed using an anti‐AGO2 antibody. The results of western blot analysis showed that endogenous DDX3 protein could be co‐precipitated with AGO2 protein in HEK293T cells (Fig. 5B), supporting the association between DDX3 and AGO2 proteins.

On the other hand, we also examined whether DDX3 interacts with Dicer in human cells. FLAG‐tagged DDX3 or PACT was transiently expressed in HEK293T cells, and cell lysates were prepared and subjected to immunoprecipitation with an anti‐FLAG antibody. The results of western blot analysis showed a strong interaction between PACT and Dicer, whereas DDX3 only slightly associates with Dicer in HEK293T cells (Fig. 5C). It appears that DDX3 may associate with the RISC complex through direct interaction with AGO2.

### DDX3 promotes cell proliferation and transformation in HEK293 cells

**Fig. 6:**
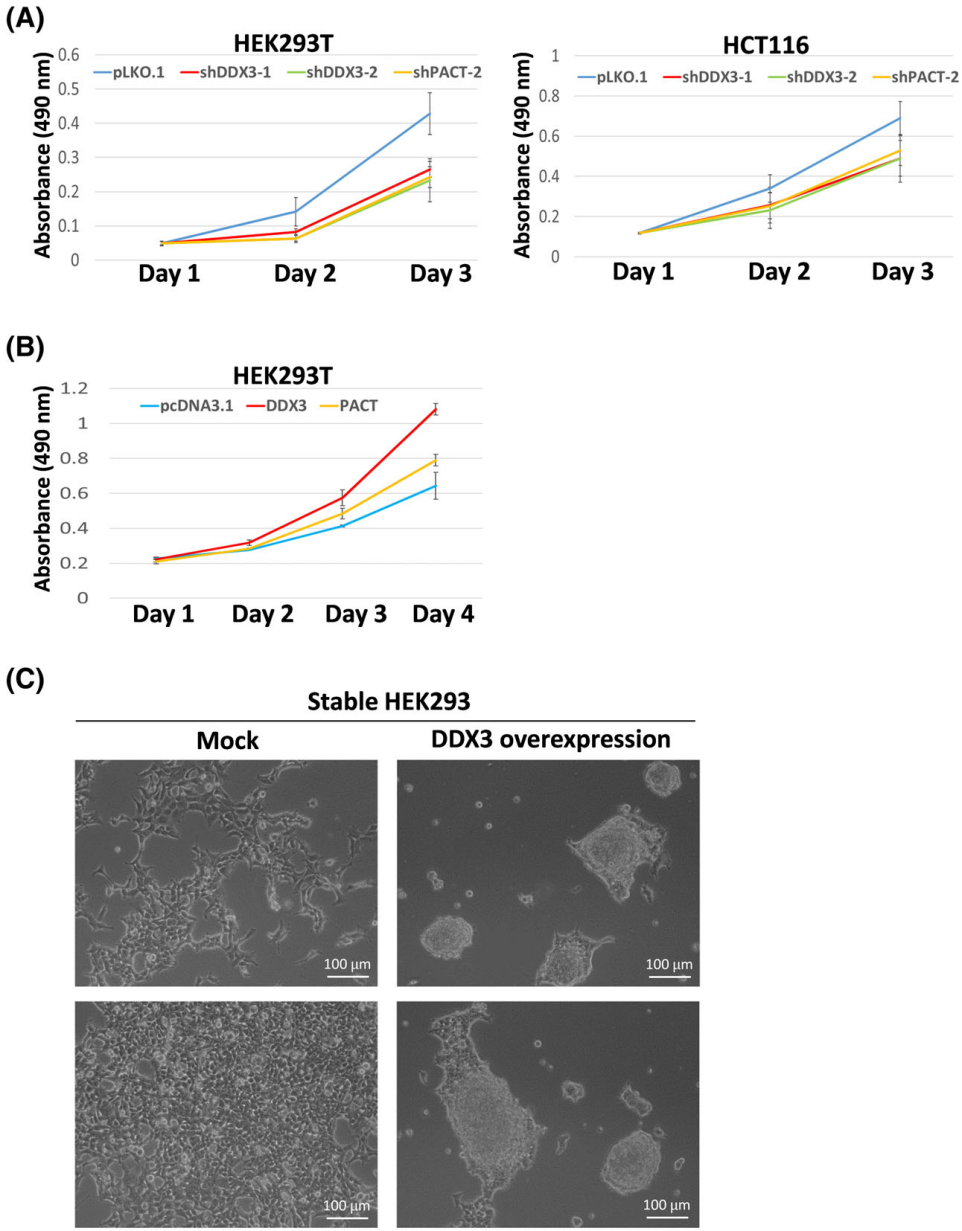
DDX3 and PACT are vital for cell proliferation and transformation. (A) HEK293T and HCT116 cells were transduced with the empty lentiviral vector (pLKO.1) or the pLKO.1 vector expressing shDDX3 or shPACT. After 2 days, infected cells were seeded at 3 × 10^3^ cells per well in a 96‐well plate. Cell proliferation was assessed by the MTS reagent for 3 days. After incubation with the MTS reagent for 1 h, the absorbance is measured at 490 nm using a plate reader. Data are shown as the mean ± SD from three independent experiments. (B) HEK293T cells were transiently transfected with the pcDNA3.1 vector or the pcDNA3.1 vector expressing FLAG‐tagged DDX3 or PACT. After 24 h, transfected cells were seeded at 3 × 10^3^ cells per well in a 96‐well plate. Cell proliferation was assessed by the MTS reagent for 4 days. After incubation with the MTS reagent for 1 h, the absorbance is measured at 490 nm using a plate reader. Data are shown as the mean ± SD from three independent experiments. (C) HEK293 cells were stably transfected with the pcDNA3.1 vector (Mock) or the pcDNA3.1 vector expressing FLAG‐tagged DDX3 (DDX3 overexpression). After selection with G418 for 2 weeks, stable cells were cultured in 6‐cm dishes. Images were acquired with an AxioObserver A1 equipped with a AxioCam MRm camera (*n* = 3, representative images shown) (Zeiss, Oberkochen, Germany).

We previously reported that DDX3 is required for cell proliferation in HeLa cells [7]. To examine the effects of DDX3 on cell proliferation in HEK293T and HCT116 cells, we performed the MTS assay to assess cell proliferation/viability in DDX3 knockdown cells compared to control cells. In addition, we also investigated the effects of PACT on cell proliferation/viability. The results of MTS assay showed that knockdown of DDX3 or PACT inhibits cell proliferation/viability (Fig. 6A), suggesting an oncogenic role for DDX3 in HEK293T and HCT116 cells. Reversely, overexpression of DDX3 or PACT increased cell proliferation/viability in HEK293T cells (Fig. 6B). This result reinforced a positive role of DDX3 and PACT in cell proliferation.

On the other hand, we also observed cell transformation in HEK293 cells that stably overexpress DDX3. The parental HEK293 cells were cultured in a monolayer and displayed intercellular contact inhibition (Fig. 6C). By contrast, DDX3‐overexpressing stable HEK293 cells cultured as multilayers, indicating the loss of intercellular contact inhibition. DDX3 promotes cell proliferation and transformation in HEK293 cells, suggesting an oncogenic role of DDX3 in cancer development.

## Discussion

DDX3 is involved in a variety of biological functions, including cell cycle [7, 16], embryonic development [16, 61], tumorigenesis and cancer progression [20, 21]. The role of DDX3 in cancers has attracted more attention in recent years. Many studies have gradually uncovered the mechanisms of DDX3 in tumorigenesis and cancer progression. However, it remains a complicated issue because DDX3 regulates gene expression at different steps, from transcription to translation, possibly including pre‐mRNA splicing and RNA export. We previously identified hundreds of candidate genes for which translation is regulated by DDX3 [7]. Pathway enrichment analysis indicates that many DDX3‐regulated genes are involved in the MAPK signaling pathway, growth hormone and the Wnt signaling pathway (data not shown). Therefore, DDX3 may affect cell growth and development by regulating the expression of many downstream target genes. In the present study, we provide evidence that DDX3 may affect cell proliferation/viability by regulating the expression of a small subset of miRNAs. miRNAs are small non‐coding RNAs that play vital roles in gene expression and regulation. The expression of miRNAs is critical in the early stages of embryonic development. Aberrant expression of miRNAs is observed in various diseases, including cancer.

Emerging evidence indicates that aberrant expression of miRNAs contributes to tumorigenesis and cancer progression. Notably, it has been reported that miR‐18a/b are significantly up‐regulated in several human cancers, such as nasopharyngeal carcinoma [46], hepatocellular carcinoma [47, 62], glioblastoma [48], osteosarcoma [49], breast cancer [45] and colorectal cancer (CRC) [63, 64]. Up‐regulation of miR‐18b is shown to be involved in the development of CRC by targeting CDKN2B [64]. The overexpression of miR‐18a is also correlated with poor clinical prognosis in CRC patients [63], and serum miR‐18a is thought to be a potential biomarker for detecting CRC [65, 66]. Overexpression of miR‐20b was also detected in CRC and breast cancer tissues [50, 51]. Both miR‐221 and its paralog miR‐222 have been described as oncogenic miRNAs in different types of cancer [55, 56, 57]. Serum miR‐221 is also considered a potential diagnostic and prognostic marker of CRC [67]. The elevated miR‐221 level significantly indicates poor survival in CRC patients. A list of differentially expressed miRNAs between colorectal cancer specimens and normal tissues was recently published [68]. The up‐regulated miRNAs include miR‐18a, miR‐18b, miR‐20b, and miR‐221, suggesting a role in cancer development. However, miR‐93 and miR‐320 have been identified as tumor‐suppressive miRNAs in CRC [69, 70, 71]. The miR‐320 family has been shown to act as a potential biomarker for cancer diagnosis, prognosis and prediction [72]. Here, we identified several oncogenic and tumor‐suppressive miRNAs as targets of DDX3. It remains to be elucidated whether these miRNAs regulated by DDX3 play an oncogenic or tumor‐suppressive role in cancer development. The expression and regulation of DDX3 for specific miRNAs may vary in different cancer cells (Fig. 3).

The role of DDX3 in miRNA biogenesis and RNAi appears to be evolutionarily conserved [32, 33, 35, 40]. The most crucial finding in the present study is that DDX3 may function in miRNA biogenesis and RNAi through translational control of PACT. Notably, we provide evidence that DDX3‐mediated translational control of PACT is also conserved in *C. elegans* and humans (Fig. 1). DDX3 specifically regulates PACT but has no noticeable effect on TRBP, which can explain why only a small subset of miRNAs is affected by DDX3 knockdown. In *C. elegans*, RDE‐4/PACT is also controlled by VBH‐1/DDX3 rather than RDE‐12/DDX4 (Fig. 1). This reinforced the specific regulation of PACT by DDX3.

On the other hand, the interaction between Belle/DDX3 and AGO proteins has been shown in *Drosophila* S2 cells [32, 33]. We also provide evidence that DDX3 is associated with AGO2 in human HEK293T cells (Fig. 5). These findings support the idea that DDX3 may play a crucial role in the RNAi pathway. A recent study showed that DDX3 modulates the expression of tumor‐suppressive miRNAs through epigenetic regulation in hepatocellular carcinoma [34]. However, the molecular mechanisms by which DDX3 regulates miRNA expression are poorly understood. It remains for further studies to elucidate the molecular mechanisms of DDX3 in miRNA biogenesis and its effect on cells.

## Conflicts of interest

The authors declare that they have no conflicts of interest.

### Peer review

The peer review history for this article is available at https://www.webofscience.com/api/gateway/wos/peer‐review/10.1002/2211‐5463.13920.

## Author contributions

M‐CL and S‐PC planned the experiments. M‐CL and Y‐LY performed most of the experiments. M‐CL, Y‐LY, C‐NC and S‐PC analyzed the data. M‐CL wrote the manuscript. J‐SY, H‐YH and S‐PC revised the manuscript.

## Supporting information


**Fig. S1.** Volcano plot of the differentially expressed miRNAs in DDX3 knockdown HEK293T cells compared to control cells.

## Data Availability

The data presented in this study are available from the corresponding author upon reasonable request.
