# Supplementary material for: DDX3 participates in miRNA biogenesis and RNA interference through translational control of PACT and interaction with AGO2
Source: FEBS Open Bio. 2024 Nov 14;15(1):180–95. doi: 10.1002/2211-5463.13920 (PMC11705417; doi:10.1002/2211-5463.13920)
Supplement: Supplementary file 1 — Fig. S1. Volcano plot of the differentially expressed miRNAs in DDX3 knockdown HEK293T cells compared to control cells. [file FEB4-15-180-s001.pdf]

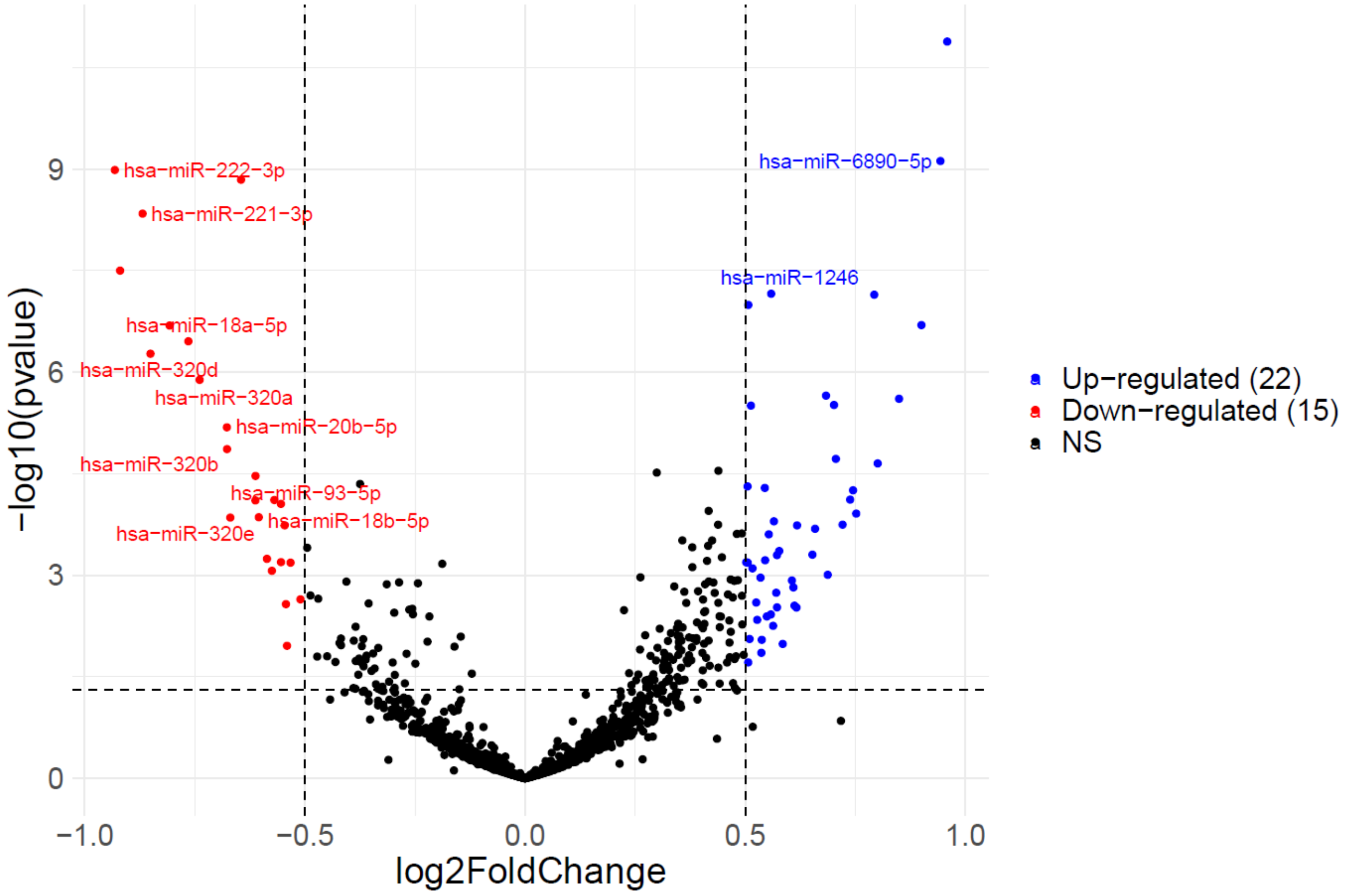

Figure S1. Volcano plot of the differentially expressed miRNAs in DDX3 knockdown HEK293T cells compared to control cells. The vertical lines correspond to 1.5-fold up and down, respectively, and the horizontal line represents a P-value of 0.05. Up-regulated miRNAs are marked in red; down-regulated miRNAs are marked in blue. The 12 miRNAs addressed by the study are labeled (n = 2).
